# Exploring the relation between childhood trauma, temperamental traits and mindfulness in borderline personality disorder

**DOI:** 10.1186/s12888-015-0573-z

**Published:** 2015-07-29

**Authors:** Matilde Elices, Juan C. Pascual, Cristina Carmona, Ana Martín-Blanco, Albert Feliu-Soler, Elisabet Ruiz, Montserrat Gomà-i-Freixanet, Víctor Pérez, Joaquim Soler

**Affiliations:** 1 Servei de Psiquiatria, Hospital de la Santa Creu i Sant Pau, Barcelona, Spain; 2 Institut d’Investigació Biomèdica - Sant Pau (IIB-Sant Pau), Centro de Investigación Biomédica en Red de Salud Mental (CIBERSAM), Madrid, Spain; 3 Departament de Psiquiatria i Medicina Legal, Universitat Autònoma de Barcelona, Barcelona, Spain; 4 Programa de Cognición. Instituto de Fundamentos y Métodos en Psicología. Facultad de Psicología, Universidad de la República, Montevideo, Uruguay; 5 Departament de Psicologia Clínica i de la Salut, Universitat Autònoma de Barcelona, Barcelona, Spain

**Keywords:** Borderline personality disorder, Mindfulness, Childhood maltreatment, Temperament

## Abstract

**Background:**

Deficits in mindfulness-related capacities have been described in borderline personality disorder (BPD). However, little research has been conducted to explore which factors could explain these deficits. This study assesses the relationship between temperamental traits and childhood maltreatment with mindfulness in BPD.

**Methods:**

A total of 100 individuals diagnosed with BPD participated in the study. Childhood maltreatment was assessed using the Childhood Trauma Questionnaire (CTQ-SF), temperamental traits were assessed using the Zuckerman-Khulman Personality Questionnaire (ZKPQ), and mindfulness capabilities were evaluated with the Five Facet Mindfulness Questionnaire (FFMQ).

**Results:**

Hierarchical regression analyses were performed including only those CTQ-SF and ZKPQ subscales that showed simultaneous significant correlations with mindfulness facets. Results indicated that neuroticism and sexual abuse were predictors of *acting with awareness*; and neuroticism, impulsiveness and sexual abuse were significant predictors of *non-judging*. Temperamental traits did not have a moderator effect on the relationship between childhood sexual abuse and mindfulness facets.

**Conclusions:**

These results provide preliminary evidence for the effects of temperamental traits and childhood trauma on mindfulness capabilities in BPD individuals. Further studies are needed to better clarify the impact of childhood traumatic experiences on mindfulness capabilities and to determine the causal relations between these variables.

## Background

Borderline personality disorder (BPD) is a severe psychiatric condition marked by a pervasive pattern of emotional dysregulation, impulsive behaviour, identity disturbances and interpersonal conflicts [1]. Previous publications have established that the transaction between biological temperamental predispositions and environmental factors contribute to the development of the disorder [2–4]. BPD has been described as an extreme and maladaptive variant of normal temperamental dimensions [1, 5, 6] such as neuroticism, impulsiveness and aggression- hostility [1, 7]. In parallel, other studies have revealed that certain contextual factors such as adverse childhood experiences are also related to BPD symptoms and its severity [8, 9]. Retrospective studies have found high rates of early traumatic experiences in BPD, comprising 30 to 90 % of BPD cases and including sexual, physical and emotional abuse [10, 11]. Traumatic experiences and neuroticism are also independent predictors of BPD severity [9]. However, it seems that the interaction between these factors (i.e. neuroticism and adverse childhood experiences) is even more complex, as it contributes not only to the severity [8] but also to the development of the disorder [12].

In recent years, psychological treatments that include mindfulness training have been increasingly used to treat individuals with BPD with and without a history of early trauma [13–15]. Moreover, it has been suggested that improvements in the patient’s mindfulness capacities are a common factor associated with the success of evidence-based therapies for BPD [16]. Mindfulness can be described as a particular way to pay attention to the present moment, in an inquiring and accepting manner, without judging or reacting to the experience [17]. The construct of mindfulness encompasses several facets [18, 19]: (1) the capacity to *observe* and notice the current experience; (2) the ability to *describe* the experience (i.e., putting into words or labelling the experience), (3) a non-judgemental and non-evaluative stance (i.e., *non-judging*), (4) *non-reactivity* to inner experience (i.e., allowing thoughts and feelings to come without getting caught up in them) and (5) *acting with awareness* (i.e., focusing on the present activity instead of behaving mechanically). The inclusion of mindfulness practices in BPD treatment is based on the idea that individuals with BPD have a deficit in certain important mindfulness capabilities and that this deficit is associated with symptoms of the disorder [20, 21]. Indeed, subjects with BPD display low levels of mindfulness compared to healthy controls and other clinical populations [22–24]. In addition, studies conducted by Wupperman and colleagues [25, 26] suggest an inverse relation between mindfulness and core characteristics of BPD, including neuroticism.

To date, research on the relationship between personality traits and the facets of mindfulness has mainly been conducted in non-clinical samples. Strong, inverse correlations between neuroticism and mindfulness [26] and between impulsivity and two facets of mindfulness (*acting with awareness* and *non-judging*) have been reported [27]. However, the influence of life-events on mindfulness capabilities has not been sufficiently explored. An exception to this is the study carried out by Michal et al. [28], in which a significant and negative correlation was found in a non-clinical population between emotional maltreatment and mindful attention and awareness. The inverse relation between certain mindfulness facets and the severity of post traumatic stress disorder (PTSD) symptoms also suggest an association between traumatic experiences and deficits in mindfulness capabilities [29–31].

Given the context described above, in this study we explore the association between temperamental traits, childhood maltreatment, and deficits in mindfulness capacities in a sample of BPD individuals. Since temperamental traits, especially neuroticism, appear to be moderators of the relationship between childhood maltreatment and psychopathology [12] as well as between childhood maltreatment and BPD severity [8], we also explored the role of temperamental traits as moderators of the association between childhood maltreatment and mindfulness. Based on the literature [27, 28], we hypothesized that some temperamental traits (specifically, neuroticism and impulsiveness) would be negatively associated with mindfulness. We also expected to find neuroticism to have a moderating effect on the association between childhood trauma and mindfulness. Research on the association between mindfulness and diverse childhood maltreatment experiences is scant. Therefore, no *a priori* hypotheses regarding the possible link between specific facets of mindfulness and different types of traumatic experiences were made.

## Method

### Participants

A total of 133 participants were recruited from the outpatient BPD unit at the Department of Psychiatry of the Hospital de la Santa Creu i Sant Pau (Barcelona, Spain). Only data from participants meeting the inclusion criteria were analysed, resulting in a final sample of 100 participants. Inclusion criteria were as follows: (1) diagnosis of BPD according to DSM-IV criteria [32] confirmed by two structured clinical interviews: Structured Clinical Interview for DSM-IV Axis II Personality Disorders (SCID-II) [33] and Revised Diagnostic Interview for Borderlines (DIB-R) [34]; (2) age between 18 and 45 years; (3) no current co-morbidity with major depression, bipolar disorder, psychotic disorders, or substance dependence; (4) no severe physical conditions or intellectual disability; and (5) no previous training in mindfulness.

### Instruments

#### Diagnostic measures

To ensure an accurate BPD diagnosis, the Spanish versions of two semi-structured clinical interviews were used. The SCID-II [33] was used to assess personality disorders according to DSM-IV criteria [32]. The SCID-II has shown adequate psychometric properties, including good reliability between interviewers (kappa: 0.85). The DIB-R [34] is a semi-structured interview to establish BPD diagnosis over the last 2 years. The cut-off point of the Spanish version is six (range: 0 to 10). Psychometric properties are good: internal consistency (Cronbach’s alpha = 0.89), sensitivity (0.81), and specificity (0.94). The convergent validity with the diagnosis of the SCID-II is moderate (kappa: 0.59).

#### Self-report questionnaires

To assess adverse childhood experiences the Childhood Trauma Questionnaire – Short Form (CTQ-SF) [35] was administered. The CTQ-SF contains 28 items and retrospectively assesses childhood abuse and neglect across five subscales described as follows: (a) sexual abuse: sexual contact or conduct between a child under 18 years of age and an adult or older person, (b) physical abuse: bodily assaults on a child by an adult or older person that entail a risk of, or resulted in, injury, (c) emotional abuse: verbal assaults, humiliating or demeaning behaviour directed toward a child by an adult or older person, (d) physical neglect: the failure of caretakers to provide for a child’s basic physical needs (food, shelter, clothing, safety, and health care), (e) emotional neglect: failure of caretakers to meet children’s basic emotional and psychological needs, including love, belonging, support and nurture. Items are rated on a 5-point Likert Scale from “never true” to “very often true”. For each sub-scale a cut-off point is provided in order to identify moderate – severe cases [36]. Across four diverse samples, all sub-scales have shown a good internal consistency (Cronbach’s alpha): sexual abuse (0.92 to 0.95), physical abuse (0.81 to 0.86), emotional abuse (0.84 to 0.89), physical neglect (0.61 to 0.78) an emotional neglect (0.85 to 0.91) [35].

Temperamental traits were evaluated with the Zuckerman–Kuhlman Personality Questionnaire (ZKPQ) [37], a self-administered 99 item scale (true/false format) based on the Alternative Five Factor Model. The ZKPQ includes five temperamental traits from a psychobiological perspective: Neuroticism–Anxiety (*N-Anx*), Impulsive– Sensation Seeking (*ImpSS*), Aggression–Hostility (*Agg-Host*), Activity (*Act*), and Sociability (*Sy*). Additionally, an infrequency scale is provided to assess inattention to the questionnaire. The Spanish version has shown good psychometric properties, including good internal consistency (Cronbach’s alpha ranging from 0.77 to 0.91), as well as satisfactory convergent, discriminant, and consensual validity [37].

The Five Facet Mindfulness Questionnaire (FFMQ) [38] was used as a mindfulness measure. The FFMQ is a 39-item questionnaire that evaluates mindfulness in five facets: (1) *observing* (noticing or attending to external and internal experiences -e.g., body sensations, thoughts or emotions-), (2) *describing* (putting words to, or labelling the internal experience), (3) *acting with awareness* (i.e., focusing on the present activity instead of behaving mechanically), (4) *non-judging* the inner experience (i.e., taking a non-evaluative stance towards thoughts or emotions), and (5) *non-reactivity* to inner experience. Participants are asked to rate the degree of concordance with each statement on a five point-likert scale ranging from one (never or very rarely true) to five (very often or always true). Cronbach’s alpha from the Spanish version ranges from 0.80 to 0.91 [39].

### Procedure

Study participants were evaluated at our unit during the years 2013 and 2014. As part of our routine assessment, diagnostic interviews were administered in two different sessions. After BPD diagnosis was confirmed, patients were invited to participate in the study. Prior to inclusion, all subjects signed an informed consent in which the study was explained and after that, completed self-report questionnaires. No remuneration (monetary or otherwise) was given for participation. The study was approved by the Ethical Research Committee at the Hospital de la Santa Creu i Sant Pau.

## Data Analysis

Descriptive statistics were used to describe the socio-demographic and clinical characteristics of the sample. Pearson correlation analyses were carried out between childhood maltreatment (CTQ-SF) and mindfulness facets (FFMQ); and between personality traits (ZKPQ) and mindfulness facets (FFMQ).

Multiple hierarchical regression analyses were used to determine the single and interactive effects of childhood trauma and temperamental variables on mindfulness facets. Associations between demographic variables (age, gender, and education level) and dispositional mindfulness were also studied by means of Pearson correlations and Student’s *t*-test to identify potential covariates for inclusion in the regression models. To create interaction terms and to account for multicolinearity, variables were mean-centred [39]. FFMQ subscales were entered as the dependent variable; temperamental traits (ZKPQ subscales) were entered in step 1, CTQ-SF subscales were included in step 2, and interaction terms (temperamental trait × type of abuse) in step 3.

## Results

### Demographic and clinical characteristics of the sample

The majority of participants were women (88 %), with an average age of 30.46 years (SD = 6.84). The mean DIB-R score suggest that the severity of BPD was high (*M* = 7.74, SD = 1.48). On the CTQ-SF subscales, the frequency of severe forms of childhood maltreatment was as follows: severe emotional abuse (63 %); severe emotional neglect (51 %); severe sexual abuse (45 %); severe physical neglect (35 %); and severe physical abuse (20 %). Additional demographic and clinical characteristics are presented in Table 1.

**Table 1:** Demographic and descriptive characteristics of the sample

	*Mean*	*(SD)*
Gender (% of females)	88	
Age (years)	30.46	(6.84)
Marital status - married or cohabitating (%)	39	
Years of education	11.95	(3.32)
Currently employed (%)	34	
DIB-R total score	7.74	(1.48)
*Childhood Trauma Questionnaire – Short Form (%)*		
Severe Emotional Abuse	63	
Severe Sexual Abuse	45	
Severe Physical Abuse	20	
Severe Emotional Neglect	51	
Severe Physical Neglect	35	
*Zuckerman-Kuhlman Personality Questionnaire*		
Neuroticism-anxiety (N-Anx)	16.00	(3.50)
Impulsive-sensation seeking (ImpSS)	10.72	(5.30)
Aggression-hostility (Agg-Host)	9.91	(4.10)
Activity (Act)	7.35	(3.62)
Sociability (Sy)	6.00	(4.12)
*Five Facet Mindfulness Questionnaire*		
Observe	24.00	(8.05)
Describe	23.15	(8.00)
Acting with awareness	20.00	(8.20)
Non-Judge	18.73	(7.57)
Non-React	15.57	(6.66)

### Correlations between childhood trauma and temperamental traits with mindfulness facets

Zero-order associations among childhood trauma (CTQ-SF), temperamental traits (ZKPQ), and mindfulness facets (FFMQ) are shown in Table 2. Negative associations were found between sexual abuse and *acting with awareness* (*r* = −.25, *p* = .03), and *non-judging* (*r* = −.27, *p* = .01). No other significant correlations were found between the other CTQ-SF subscales and mindfulness facets.

**Table 2:** Associations between childhood trauma, temperamental traits and mindfulness facets

	FFMQ				
	Observing	Describing	Acting with Awareness	Non-Judging	Non- Reactivity
CTQ – SF subscales					
Emotional Abuse	.16	−.01	−.05	−.10	−.03
Physical Abuse	.17	.00	.06	−.06	−.06
Sexual Abuse	−.03	.09	−.25*	−.27*	−.10
Emotional Neglect	.89	−.08	.01	.07	.11
Physical Neglect	.17	.04	−.08	−.17	.02
ZKPQ subscales					
ImpSS	.15	−.07	−.19	−.27**	−.05
N-Anx	.03	−.17	−.49**	−.53**	−.23*
Agg-Host	−.11	−.01	−.01	−.04	.03
Act	.20	.06	.00	−.11	.07
Sy	.12	.11	.01	−.00	.13

*CTQ-SF* Childhood Trauma Questionnaire – Short Form, *ZKPQ* Zuckerman Kuhlman Personality Questionnaire, *ImpSS* impulsive-sensation seeking, *N-Anx* neuroticism-anxiety, *Agg-Host* aggression-hostility, *Act* activity, *Sy* sociability, *FFMQ* Five Facet Mindfulness Questionnaire

*p < .05; **p < .01

*N-Anx* correlated significantly and inversely with several mindfulness facets: *acting with awareness* (*r* = −.49, *p* = <.001), *non-judging* (*r* = −.53, *p* < .001=), and *non-reactivity* (*r* = −.23, *p* = <.001). In addition, significant but weaker correlations were found between *ImpSS* and *non-judging* (*r* = −.27, *p* = .01).

### Predictive effect of childhood trauma and temperamental traits on mindfulness facets

Given that no associations were found between demographic variables (age, gender, and education level) and any of the mindfulness facets, these variables were not included in the regression models. Moreover, since our primary interest was to study the effects of both childhood maltreatment and personality variables on mindfulness capacity, regression analyses included only those CTQ-SF and ZKPQ subscales that showed simultaneous significant Pearson correlations (*p* < .05) with mindfulness. The first model included *non-judging* as the dependent variable, *N-Anx* and *ImpSS* in step 1, sexual abuse in step 2, and interaction terms (*N-Anx* × sexual abuse and *ImpSS* × sexual abuse) in step 3. Temperamental traits, *N-Anx* and *ImpSS,* were significant predictors of *non-judging,* explaining 32 % of variance. When sexual abuse was added to the model, this increased the percentage of explained variance to 35 %. Interaction effects did not attain significance in the model. The second model included *acting with awareness* as the dependent variable, *N-Anx* in step 1, sexual abuse in step 2, and the interaction term (*N-Anx* × sexual abuse) in step 3. *N-Anx* and sexual abuse were significant predictors of acting with awareness, explaining 27 % of the variance. No significant effect of the interaction between *N-Anx* and sexual abuse was found. Results of the regression analyses are shown in Table 3.

**Table 3:** Hierarchical multiple regression analyses predicting FFMQ facets: non-judging (column A) and acting with awareness (column B)

		A						B					
		Non-Judging							Acting with awareness				
		*B*	*B se*	*β*	*R* ^*2*^	Δ*R*^*2*^			*B*	*B se*	*β*	*R* ^*2*^	Δ*R*^*2*^
	Predictor							Predictor					
Step 1	N-Anx	−.95	.19	−.45***	.32	.33***	Step 1	N-Anx	−1.07	.22	−.47***	.24	.25***
	ImpSS	−.44	.16	−.25**									
Step 2	Sexual abuse	−.29	.13	−.19*	.35	.03*	Step 2	Sexual abuse	−.31	.16	−.19*	.27	.03*
Step 3	Sexual abuse x N-Anx	−.09	.07	−.15	.35	.02	Step 3	Sexual abuse x N-Anx	−.00	.08	−.00	.25	.00
	Sexual abuse x ImpSS	.01	.03	.04									

R^2^ = Adjusted R^2^

*ImpSS* impulsive-sensation seeking, *N-Anx* neuroticism-anxiety

*p < .05, **p < .01,***p < .001

## Discussion

The present study sought to investigate the association between temperamental traits, different types of childhood trauma, and various facets of mindfulness in a sample BPD patients. Our results indicate that there is a significant and negative association between *N-Anx* and several mindfulness facets: *acting with awareness, non-judging* and *non-reactivity* and between impulsiveness and *non-judging*. Additionally, five different types of childhood maltreatment were assessed, but only sexual abuse appears to be related with mindfulness deficits, having a negative impact on *acting with awareness* and increasing the judgmental stance towards inner and outer experiences. Based on our findings, it appears that temperamental traits might not play a role in moderating the relationship between a history of sexual abuse and mindfulness deficits. Overall, the present study indicates that temperamental traits might have a greater impact on mindfulness capacities than early traumatic experiences in BPD patients.

As suggested by previous work, our results indicate that neuroticism and impulsivity have a significant and negative association with mindfulness facets [20, 27, 28, 40]. However to the best of our knowledge, ours is the first study to test these associations in a BPD sample. *N-Anx* was a significant predictor of *acting with awareness* and, together with *ImpSS*, it was also a significant predictor of *non-judging*, indicating that higher scores on these traits are related to greater difficulties in being present-oriented and comprise a more judgemental and evaluative stance towards the experiences, consistent with BPD characteristics [21, 41].

In congruence with a previous study [42], it seems that having a history of childhood sexual abuse compromises the ability to *act with awareness*. Awareness difficulties can be explained by the presence of trauma-related thoughts and memories. In fact, a study involving adult sexual abuse survivors who underwent mindfulness treatment reported a decrease in re-experiencing and avoidance of numbing symptoms and an increase in mindfulness attention and awareness, suggesting a possible relationship between these two aspects [42]. Our results also indicate an association between judgemental information processing and sexual abuse. However, and considering that a history of sexual abuse only increased the explained variance in the regression analyses by a small amount, it seems that –at least in our sample- the impact of temperamental traits on mindfulness capacity is stronger than the influence of sexual abuse. In any case, the association between mindfulness facets and sexual abuse highlights the relevance of addressing mindfulness deficits during the treatment of BPD patients with traumatic histories [13, 14, 43]. Some interventional approaches combine exposure techniques (targeting direct experience processing and fostering awareness of the present experience) with mindfulness practice (to increase acceptance and diminish judgemental stances) [13, 43], and -in light of the present results- this approach would seem to be relevant to the treatment of trauma. Our findings suggest that not all types of childhood maltreatment are related to mindfulness facets in subjects with BPD. Even though sexual abuse may entail more severe and far-reaching consequences for BPD patients than other forms of abuse [11], the lack of a significant association in our study between other types of maltreatment and mindfulness facets was unexpected. Nevertheless, other studies [30, 31, 44] have found negative associations between mindfulness and PTSD symptoms and one study reported an inverse association between emotional maltreatment and awareness [28]. This difference between our study and others may be due to methodological or patient sample differences; however, further research in larger samples will be needed to better assess the relationship between childhood maltreatment and mindfulness facets.

Considering previous research on the moderating role of neuroticism in the psychopathology and BPD severity [8, 12], we hypothesized that temperamental traits would play a role in moderating the relationship between traumatic experiences and mindfulness. Unexpectedly, we did not find any moderating effect of temperamental traits on *acting with awareness* or *non-judging*.

The present results should be interpreted in the context of the study limitations. The most important limitation is the cross-sectional design, which prevents us from reaching any conclusion about the direction of the associations. Second, the use of self-report measures to assess the variables (particularly childhood trauma) could have biased the results. Third, a comparison between the BPD group and other clinical populations would have been valuable to determine if the associations found in this study are specific to BPD or not.

## Conclusions

Our results provide preliminary evidence demonstrating an association between certain temperamental traits and childhood experiences with mindfulness in a sample of individuals with BPD. It seems that, in individuals with BPD, mindfulness deficits may be more closely associated with high levels of neuroticism and impulsivity rather than early traumatic experiences. Although these findings should be taken with caution, it appears that mindfulness-based interventions could offer a valuable approach to treating emotionally-dysregulated individuals with a history of trauma. This approach might not only increase acceptance, but may also offer a possible pathway to increase awareness while decreasing avoidance symptoms. More research is needed to clarify the relationship between early traumatic experiences and mindfulness, as well as to determine the mechanism underlying the efficacy of mindfulness-based treatments for individuals with a history of traumatic experiences.
