# Correction to: Exploring the relation between childhood trauma, temperamental traits and mindfulness in borderline personality disorder

**DOI:** 10.1186/s12888-018-1834-4

**Published:** 2018-09-04

**Authors:** Matilde Elices, Juan C. Pascual, Cristina Carmona, Ana Martín-Blanco, Albert Feliu-Soler, Elisabet Ruiz, Montserrat Gomà-i-Freixanet, Víctor Pérez, Joaquim Soler

**Affiliations:** 1 Servei de Psiquiatria, Hospital de la Santa Creu i Sant Pau, Barcelona, Spain; 2 Institut d’Investigació Biomèdica - Sant Pau (IIB-Sant Pau), Centro de Investigación Biomédica en Red de Salud Mental (CIBERSAM), Madrid, Spain; 3 Departament de Psiquiatria i Medicina Legal, Universitat Autònoma de Barcelona, Barcelona, Spain; 4 Programa de Cognición. Instituto de Fundamentos y Métodos en Psicología. Facultad de Psicología, Universidad de la República, Montevideo, Uruguay; 5 Departament de Psicologia Clínica i de la Salut, Universitat Autònoma de Barcelona, Barcelona, Spain

## Correction

In the original publication of this article [1] the funding acknowledgement for grant **“PI13/00134, ERDF Funds”** was missing. In this correction article the updated funding acknowledgement is shown. The missing funding acknowledgement has been indicated in **bold**.

- This study was supported by Centro de Investigación Biomédica en Red de Salud Mental (CIBERSAM) and by a grant from Instituto de Salud Carlos III (PI10/00253, PI11/00725 and **PI13/00134, ERDF Funds**). The first author (ME) was supported by a grant from Facultad de Psicología (UdelaR). JS was supported by PROMOSAM: Investigación en procesos, mecanismos y tratamientos psicológicos para la promoción de la salud mental, (Red de Excelencia PSI2014–56303-REDT) founded by Ministerio de Economía y Competitividad (2014).
